# The Journal of Inflammation

**DOI:** 10.1186/1476-9255-1-1

**Published:** 2004-09-27

**Authors:** Neville A Punchard, Cliff J Whelan, Ian Adcock

**Affiliations:** 1 Division of Science, University of Luton, Luton, UK; 2 Thoracic Medicine, National Heart and Lung Institute, Imperial College of Science, Technology and Medicine, London, UK

## Abstract

Welcome to the *Journal of Inflammation*, the first open-access, peer-reviewed, online journal to focus on all aspects of the study of inflammation and inflammatory conditions. While research into inflammation has resulted in great progress in the latter half of the 20th century, the rate of progress is rapidly accelerating. Thus there is a need for a vehicle through which this very diverse research can be made readily available to the scientific community. The *Journal of Inflammation*, a peer reviewed journal, provides the ideal vehicle for such rapid dissemination of information. The *Journal of Inflammation *covers the full range of underlying cellular and molecular mechanisms involved, not only in the production of the inflammatory responses but, more importantly in clinical terms, in the healing process as well. This includes molecular, cellular, animal and clinical studies related to the study of inflammatory conditions and responses, and all related aspects of pharmacology, such as anti-inflammatory drug development, trials and therapeutic developments, etc. All articles published in the Journal of Inflammation are immediately listed in PubMed, and access to published articles is universal and free through the internet.

## Introduction

Based on visual observation, the ancients characterised inflammation by five cardinal signs, namely redness (*rubor*), swelling (*tumour*), heat (*calor*; only applicable to the body' extremities), pain (*dolor*) and loss of function (*functio laesa*). The first four of these signs were named by Celsus in ancient Rome (30–38 B.C.) and the last by Galen (A.D 130–200) [1]. More recently, inflammation was described as "the succession of changes which occurs in a living tissue when it is injured provided that the injury is not of such a degree as to at once destroy its structure and vitality" [2], or "the reaction to injury of the living microcirculation and related tissues [3]. Although, in ancient times inflammation was recognised as being part of the healing process, up to the end of the 19^th ^century, inflammation was viewed as being an undesirable response that was harmful to the host. However, beginning with the work of Metchnikoff and others in the 19^th ^century, the contribution of inflammation to the body's defensive and healing process was recognised [1]. Furthermore, inflammation is considered the cornerstone of pathology in that the changes observed are indicative of injury and disease.

The classical description of inflammation accounts for the visual changes seen. Thus, the sensation of heat is caused by the increased movement of blood through dilated vessels into the environmentally cooled extremities, also resulting on the increased redness (due to the additional number of erythrocytes passing through the area). The swelling (oedema) is the result of increased passage of fluid from dilated and permeable blood vessels into the surrounding tissues, infiltration of cells into the damaged area, and in prolonged inflammatory responses deposition of connective tissue. Pain is due to the direct effects of mediators, either from initial damage or that resulting from the inflammatory response itself, and the stretching of sensory nerves due to oedema. The loss of function refers to either simple loss of mobility in a joint, due to the oedema and pain, or to the replacement of functional cells with scar tissue.

Today it is recognised that inflammation is far more complex than might first appear from the simple description given above and is a major response of the immune system to tissue damage and infection, although not all infection gives rise to inflammation. Inflammation is also diverse, ranging from the acute inflammation associated with *S. aureus *infection of the skin (the humble boil), through to chronic inflammatory processes resulting in remodeling of the artery wall in atherosclerosis; the bronchial wall in asthma and chronic bronchitis, and the debilitating destruction of the joints associated with rheumatoid arthritis.

These processes involve the major cells of the immune system, including neutrophils, basophils, mast cells, T-cells, B-cells, etc. However, examination of a range of inflammatory lesions demonstrates the presence of specific leukocytes in any given lesion. That is, the inflammatory process is regulated in such a way as to ensure the appropriate leukocytes are recruited. These events are controlled by a host of extracellular mediators and regulators, including cytokines, growth factors, eicosanoids (prostaglandins, leukotrines, etc), complement and peptides. In fact, it is the discovery of many of these mediators over the past 20 years that has increased our understanding of the regulation of the inflammatory process whilst, at the same time, revealing its complexity. These extracellular events are matched by equally complex intracellular signalling control mechanisms, with the ability of cells to assemble and disassemble an almost bewildering array of signalling pathways as they move from inactive to dedicated roles within the inflammatory response and site.

Which cells and mediators come into play depends on wide range of factors. These include: what stage the process of inflation is at; the initiating event, i.e. type of pathogen, auto-immune, chemical or physical injury, etc.; the tissue or organ involved; whether the inflammation is of an acute, resolving form or chronic, non resolving or long-lasting type; whether formation of granuloma is involved, or whether scarring results.

The role of inflammation as a healing, restorative process, as well as its aggressive role, is also more widely recognised today. Inflammation is now considered as the full circle of events, from initiation of a response, through the development of the cardinal signs above, to healing and restoration of normal appearance and function of the tissue or organ. However, in certain conditions there appears to be no resolution and a chronic state of inflammation develops that may last the life of the individual. Such conditions include the inflammatory disorders rheumatoid arthritis, osteoarthritis, inflammatory bowel diseases, retinitis, multiple sclerosis, psoriasis and atherosclerosis.

In order to study inflammation a multidisciplinary approach is necessary. Classically, it has required the study of the immune system, in order to understand the events involved in initiating and maintaining inflammatory conditions. Today it is recognised that the underlying genetics and molecular biology basis to cellular responses are also important in order to identify genetic predisposition to inflammatory diseases, while pharmacological studies are necessary to identify targets and develop novel treatments to bring relief from chronic life-threatening inflammatory conditions. Thus research into inflammation includes not only the study of immunological and cellular responses involved but also the pharmacological process involved in drug development.

Many of the drugs used in the treatment of inflammatory conditions, predate our current understanding of the biochemical processes involved in the disease. Traditionally, the standard treatments for rheumatoid arthritis has been to use a non-steroidal anti-inflammatory drug (NSAID), such as aspirin, for pain relief and to use corticosteroids or even disease-modifying anti-rheumatic drugs in an attempt to reduce other symptoms of the disease.

For many years the pharmaceutical industry attempted to develop NSAIDs which shared the therapeutic action of aspirin but which did not cause the main adverse event, namely gastric ulceration. This research led to the development of indomethacin, the fenamates, ibuprofen and many others. However, while all these drugs had clinical utility they also eroded the gastric mucosa. In addition, this research also led to the development of some of the animal models still used in arthritis research today, such as carrageenin oedema [4] and adjuvant arthritis [5,6]).

The development of NSAIDs, with reduced potential to cause gastric ulcers, was finally realised with the demonstration that clinically useful NSAIDs inhibited the enzyme cyclo-oxygenase [7], which was also present in the gastric mucosa. The finding that cyclo-oxygenase present in inflammatory lesions (COX2) was distinct from that found in the stomach (COX1) led to the development of selective COX2 inhibitors, such as celecoxib. These drugs provide relief from many of the symptoms of arthritis but have a reduced potential to cause gastric ulceration [8]. The differential responsiveness to these, and other, therapeutic agents and, indeed, the induction of the inflammatory response in some patients with asthma by aspirin, has led to the concept of pharmacogenomics to understand individual drug sensitivities with a view to producing therapy tailored to the individual.

Similarly, glucocorticoids are widely used in the treatment of inflammation. Unlike the NSAIDs these agents do not relieve pain but reduce inflammation by inhibiting leukocyte function. The active ingredient responsible for the anti-inflammatory activity of adrenal cortex extracts was discovered in the 1940s. This led to the use of cortisol as an anti-inflammatory and the development of potent synthetic agents typified by dexamethasone. However, because cortisol, and synthetic glucocorticoids, produce their therapeutic action at supra-physiological concentrations, adverse effects, such as suppression of the HPA-axis and Cushingoid changes are inevitable. Many of these adverse effects can be avoided by giving glucocorticoids topically. This has led to the development of inhaled glucocorticoids for the treatment of inflammatory diseases of the respiratory tract and steroid containing creams for the treatment of skin inflammation. However, applying this approach to the treatment of rheumatoid arthritis necessitates the use of intra-articular injection. Thus, there is a clear unmet medical need for a drug that provides relief from the symptoms of inflammation but can be given systemically.

The fact that a large number of patients with severe chronic inflammatory disease fail to respond to conventional systemic or topical therapy resulting in a huge clinical and socio-economic burdon underlies the need to develop novel therapies.

Thus, modern research has used molecular techniques to identify which genes are regulated by glucocorticoid receptors in an attempt to identify novel therapeutic targets. This work has attempted to fine tune the immune system through use of agents that inhibit specific pathways and mediators rather than to suppress immune cell activity. Examples of such approaches include the development of anti-TNFa therapies, anti adhesion molecule therapies and inhibitors of cytokines believed to be pivotal in a given pathology [9]. Furthermore, inhibitors of selective pro-inflammatory intracellular signalling pathways are currently in use e.g. cyclsporin or under development e.g. NF-κB, p38 MAPK and PDE4 inhibitors [10-17]. As we understand more about the complexity of the inflammatory response and the actions of the currently available drugs the value of particular clusters of targets becomes apparent. However, the success of anti-TNFα therapy in RA underlines the importance of understanding/discovering the initial driver(s) of the inflammatory response in individual diseases and patients.

While research into inflammation has resulted in great progress in the latter half of the 20th century, we recognise that the rate of progress is accelerating. Furthermore, it is our perception that there is a need for a vehicle through which this very diverse research can readily be made available to the scientific community. Thus, we have initiated the creation of the *Journal of Inflammation*, a peer reviewed journal which will enable such information to be rapidly disseminated.

## What is the Journal of Inflammation?

*Journal of Inflammation *will consider for publication all forms of original research articles, reviews, commentaries, hypothesis, meeting abstracts (by special arrangement) and comments on all aspects of inflammation. The *Journal of Inflammation *considers the term inflammation today to include the full range of underlying cellular and molecular mechanisms involved, not only in the production of the inflammatory responses but, more importantly in clinical terms, in the healing process as well. Thus the Journal covers molecular, cellular, animal and clinical studies, and related aspects of pharmacology, such as anti-inflammatory drug development, trials and therapeutic developments, etc. It will also consider publication of negative findings.

*Journal of Inflammation *aims to become the leading Internet journal (I-journal) on inflammation and, as online journals eventually replace traditionally published print journals over the next decade, the main archived journal on inflammation. The *Journal of Inflammation *has an open peer-review process, aimed at improving the accountability of peer review and giving reviewers credit for the work they do. This means that we ask reviewers to agree to their named report being passed on to the authors. Each article submitted is reviewed by at least two independent reviewers, with the aim of reaching a decision on publication within 14 days of initial receipt.

*Journal of Inflammation *is edited by Drs Neville Punchard and Cliff Whelan, and is supported by an international Advisory Board of Associate Editors and an Editorial Board drawn from the Academic and Industrial research community.

## Open Access

*Journal of Inflammation *is an Open Access, peer-reviewed online journal offering rapid world-wide access to research into all aspects inflammation. Open Access policy changes the way in which articles are published. First, all articles become freely and universally accessible online, and so an author's work can be read by anyone at no cost. This rapid and immediate access to research findings in inflammation will aid in promoting the dynamic and productive dialogue between industrial and academic members of the inflammation research community that plays such an important part in the development of future generations of anti-inflammatory therapies.

Second, the authors hold copyright for their work and grant anyone the right to reproduce and disseminate the article, provided that it is correctly cited and no errors are introduced [18]. Third, a copy of the full text of each Open Access article is permanently archived in an online repository separate from the journal. *Journal of Inflammation's *articles are archived in PubMed Central [19], the US National Library of Medicine's full-text repository of life science literature, and also in repositories at the University of Potsdam [20] in Germany, at INIST [21] in France and in e-Depot [22], the National Library of the Netherlands' digital archive of all electronic publications.

Open Access has four broad benefits for science and the general public. First, authors are assured that their work is disseminated to the widest possible audience, given that there are no barriers to access their work. This is accentuated by the authors being free to reproduce and distribute their work, for example by placing it on their institution's website. It has been suggested that free online articles are more highly cited because of their easier availability [23]. Second, the information available to researchers will not be limited by their library's budget, and the widespread availability of articles will enhance literature searching [24]. Third, the results of publicly funded research will be accessible to all taxpayers and not just those with access to a library with a subscription. As such, Open Access could help to increase public interest in, and support of, research. Note that this public accessibility may become a legal requirement in the USA if the proposed Public Access to Science Act is made law [25]. Fourth, a country's economy will not influence its scientists' ability to access articles because resource-poor countries (and institutions) will be able to read the same material as wealthier ones (although creating access to the internet is another matter [26]).

## Competing interests

Dr Neville A. Punchard is also a Section Editor for Current Opinion in Investigational Drugs.
